# Supplementary material for: Parallel single-cell metabolic analysis and extracellular vesicle profiling reveal vulnerabilities with prognostic significance in acute myeloid leukemia
Source: Nat Commun. 2024 Dec 30;15:10878. doi: 10.1038/s41467-024-55231-9 (PMC11685939; doi:10.1038/s41467-024-55231-9)
Supplement: Supplementary file 1 — Supplementary Information [file 41467_2024_55231_MOESM1_ESM.pdf]

Supplementary Figures and Tables for

# ‘Parallel single-cell metabolic analysis and extracellular vesicle profiling reveal vulnerabilities with prognostic significance in acute myeloid leukemia’

*Forte D et al.*

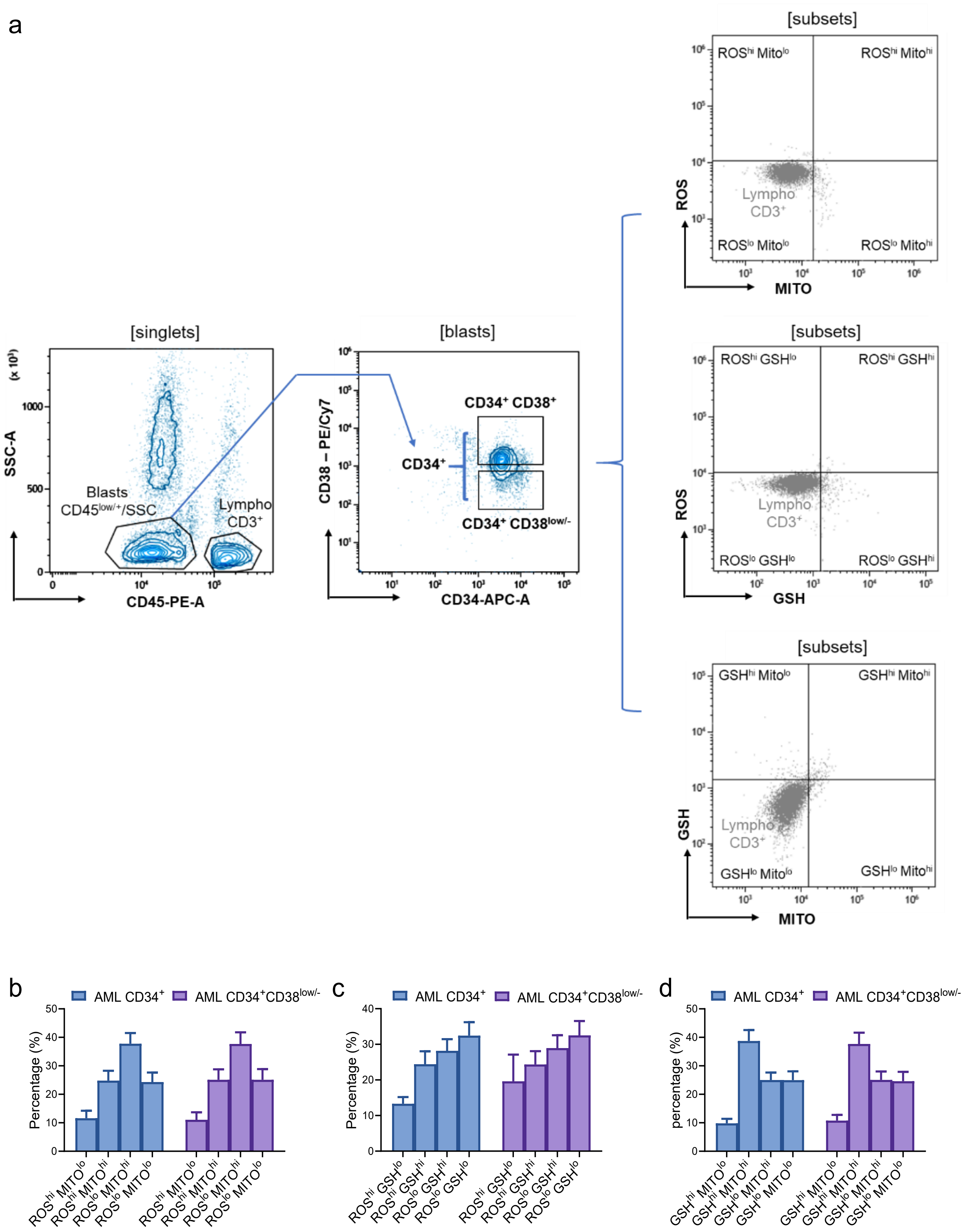

a
[singlets]
SSC-A
(x 10³)
1000
500
0
Blasts
CD45low/+/SSC
Lympho
CD3+
0
10⁴
10⁵
CD45-PE-A
[blasts]
CD38 – PE/Cy7
CD34+ CD38+
CD34+
CD34+ CD38low/-
CD34-APC-A
[subsets]
ROShi Mitolo
ROShi Mitohi
ROSlo Mitolo
ROSlo Mitohi
ROS
MITO
Lympho
CD3+
[subsets]
ROShi GSHlo
ROShi GSHhi
ROSlo GSHlo
ROSlo GSHhi
ROS
GSH
Lympho
CD3+
[subsets]
GSHhi Mitolo
GSHhi Mitohi
GSHlo Mitolo
GSHlo Mitohi
GSH
MITO
Lympho
CD3+
b
AML CD34+
AML CD34+CD38low/-
Percentage (%)
ROShi MITOlo
ROShi MITOhi
ROSlo MITOhi
ROSlo MITOlo
c
AML CD34+
AML CD34+CD38low/-
Percentage (%)
ROShi GSHlo
ROShi GSHhi
ROSlo GSHhi
ROSlo GSHlo
d
AML CD34+
AML CD34+CD38low/-
percentage (%)
GSHhi MITOlo
GSHhi MITOhi
GSHlo MITOhi
GSHlo MITOlo

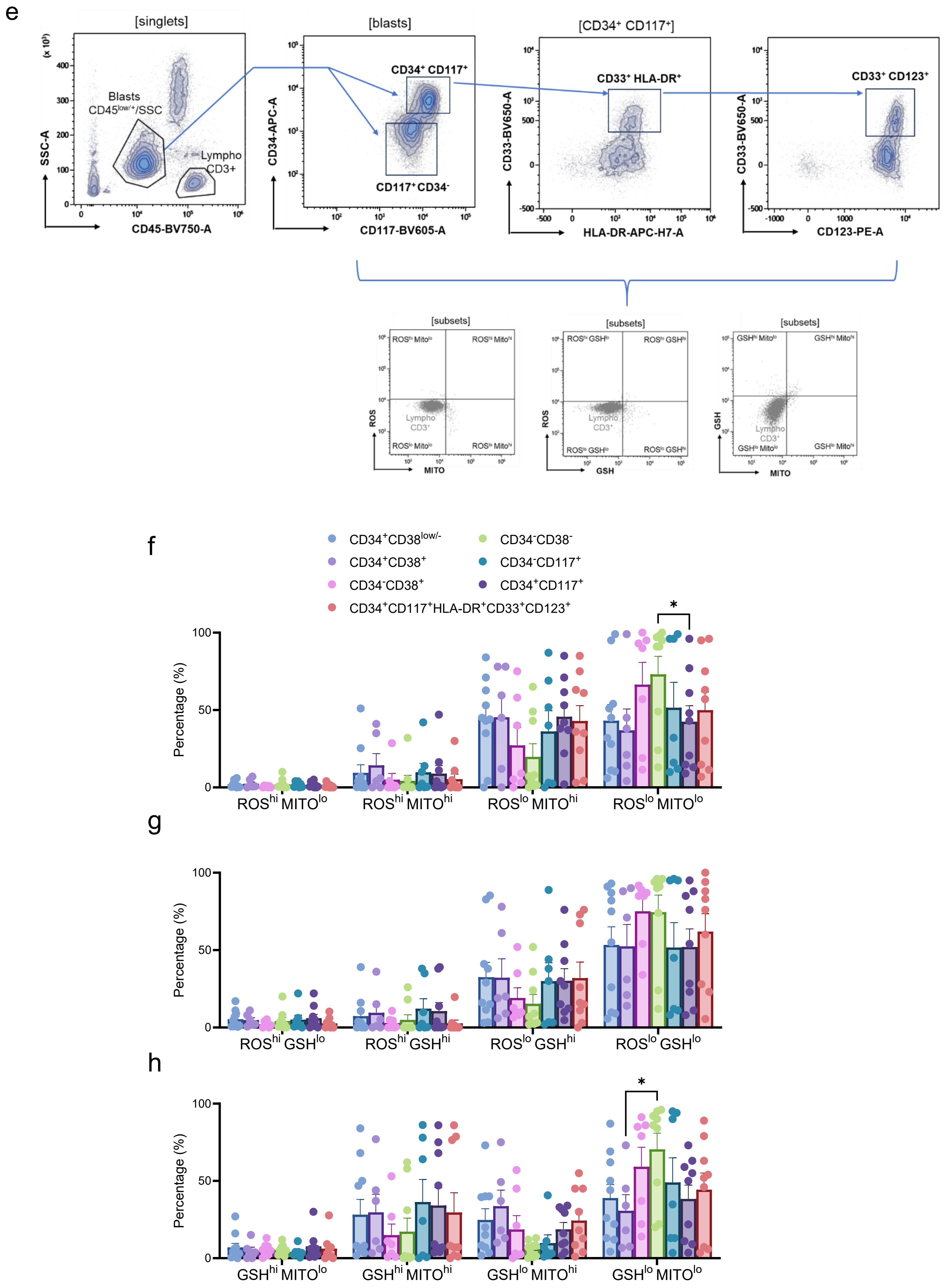
e
[singlets]
Blasts CD45low/+/SSC
Lympho CD3+
SSC-A
CD45-BV750-A
[blasts]
CD34+ CD117+
CD117+CD34-
CD34-APC-A
CD117-BV605-A
[CD34+ CD117+]
CD33+ HLA-DR+
CD33-BV650-A
HLA-DR-APC-H7-A
CD33+ CD123+
CD123-PE-A
[subsets]
ROS
MITO
GSH
f
CD34+CD38low/-
CD34+CD38+
CD34-CD38+
CD34+CD117+HLA-DR+CD33+CD123+
CD34-CD38-
CD34-CD117+
CD34+CD117+
Percentage (%)
ROShi MITOlo
ROShi MITOhi
ROSlo MITOhi
ROSlo MITOlo
g
ROShi GSHlo
ROShi GSHhi
ROSlo GSHhi
ROSlo GSHlo
h
GSHhi MITOlo
GSHhi MITOhi
GSHlo MITOhi
GSHlo MITOlo

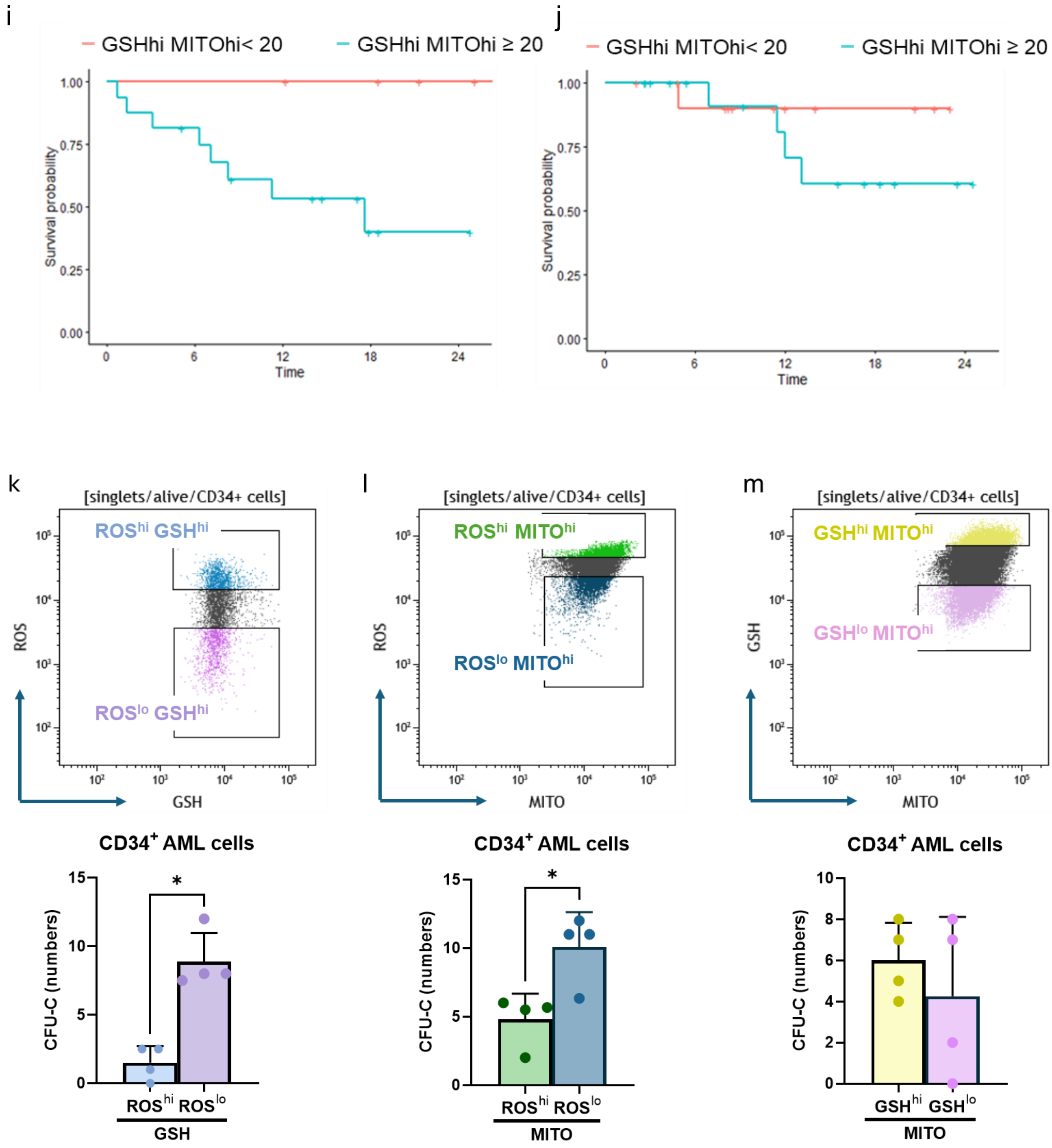


**Supplementary Figure 1**

a) Gating strategy 1. Blast populations were identified by CD45$^{low/+}$/SSC gating strategy to define and analyze mainly CD34$^{+}$ stem cells, immature CD34$^{+}$CD38$^{low/-}$ stem cells and CD34$^{+}$CD38$^{+}$ progenitor cells. For redox metabolic analysis, the CD34$^{+}$ leukemic subsets were analyzed in combination with CellROX (ROS), MitoTracker CMXRos (MITO), and Thiol Tracker (GSH) using two-by-two gating on CD3$^{+}$ cells as a reference marker (lympho CD3$^{+}$ gate). Redox metabolic profile of CD34$^{+}$ stem cells, immature CD34$^{+}$CD38$^{low/-}$ stem cells cells according to two-by-two combinations: ROS/GSH/MITO. Column graphs for profiling AML PB CD34$^{+}$ cells in comparison to paired more immature CD34$^{+}$CD38$^{low/-}$ cells according to ROS/MITO (b), ROS/GSH (c), GSH/MITO (d) (n = 59). No statistical significance was reported by two-way ANOVA comparing all columns. e) Gating strategy 2. Blast populations were identified by CD45$^{low/+}$/SSC gating strategy to define and analyze immature and progenitor leukemic cells (CD34$^{+}$ and/or, CD117$^{+}$), myeloid cells (CD33$^{+}$, HLA-DR$^{+}$), and primitive LSC (CD123$^{+}$). For redox metabolic analysis, the leukemic subsets were analyzed in combination with CellROX (ROS), MitoTracker CMXRos (MITO), and Thiol Tracker (GSH) using two-by-two gating based on CD3$^{+}$ cells as reference marker (lympho CD3$^{+}$ gate). Column graphs for profiling the

following leukemic subsets: $CD34^+CD38^{low/-}$, $CD34^+CD38^+$, $CD34^-CD38^+$, $CD34^-CD38^-$, $CD34^-CD117^+$, $CD34^+CD117^+$, $CD34^+CD117^+HLA\text{-}DR^+CD33^+CD123^+$ according to ROS/MITO (f), ROS/GSH (g), GSH/MITO (h) (n = 10). Two-way, repeated measure, mixed-effects ANOVA with Geisser-Greenhouse was performed to detect significant differences between subsets. The Kaplan-Meier survival analyses based on the $GSH^{hi}$ $MITO^{hi}$ cut-off point of 20% according to therapy. i) The OS rates of patients with lower $GSH^{hi}$ $MITO^{hi}$ values (<20%) was significantly higher than those with higher $GSH^{hi}$ $MITO^{hi}$ values (≥20%) in AML patients receiving chemotherapy. The log-rank test was used to evaluate significance (n = 21, $p = 0.04$). j) No significant correlations were reported in OS of AML receiving no chemotherapy (n = 28, $p = 0.35$).
k-m) On the top, representative dot plots illustrating the gating strategy used for cell sorting. On the bottom: k) CFU counts in $CD34^+$ AML cells sorted based on high GSH content, comparing ROS low (purple column) to ROS high (cyan column) fractions ($p = 0.03$). l) CFU counts in $CD34^+$ AML cells with high mitochondrial functionality, comparing ROS low (cerulean column) to ROS high (green column) fractions ($p = 0.03$). m) CFC counts in $CD34^+$ AML cells sorted for high mitochondrial functionality, comparing GSH low (pink column) to GSH high (yellow column) fractions (n = 4 independent experiments). Significant differences were reported using the Mann–Whitney test for unpaired samples with (*) $p < 0.05$ considered significant. Data are presented as mean values ± SEM.

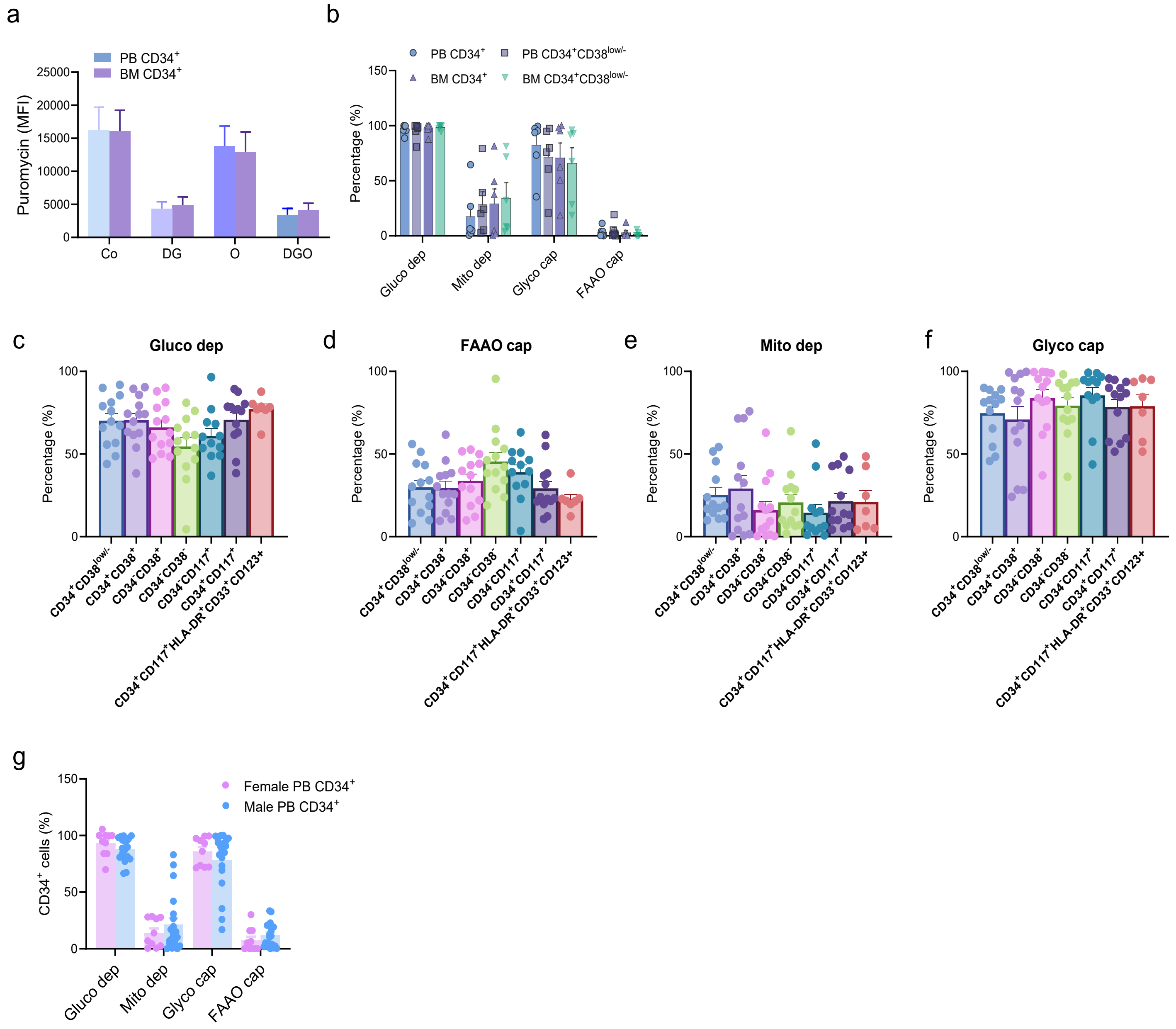


## Supplementary Figure 2

Metabolic profile of AML LSCs using SCENITH$^{TM}$. a) Translation level (anti-Puro gMFI) by puromycin value after inhibition of metabolic pathways with Co (control), 2-Deoxy-D-Glucose (DG), Oligomycin A (O) or both (DGO) (AML patients, n = 24) comparing paired AML CD34$^{+}$ from PB versus BM. b) Comparison between AML CD34$^{+}$ ± CD38$^{low/-}$ derived from PB versus BM for glucose dependence (Gluco dep), mitochondria dependence (Mito dep), or glycolytic capacity (Glyco cap) and fatty acid and amino acid oxidation (FAAO cap) expressed in percentage (n = 6). c-f) Comparison between leukemic cell subsets derived from PB for glucose dependence (Gluco dep; c), fatty acid and aminoacid oxidation (FAAO cap; d), mitochondria dependence (Mito dep; e) and glycolytic capacity (Glyco cap; f) expressed in percentage for CD34$^{+}$CD38$^{low/-}$, CD34$^{+}$CD38$^{+}$, CD34$^{-}$CD38$^{+}$, CD34$^{-}$CD38$^{-}$, CD34$^{-}$CD117$^{+}$, CD34$^{+}$CD117$^{+}$ and CD34$^{+}$CD117$^{+}$HLA-DR$^{+}$CD33$^{+}$CD123$^{+}$ subsets in fresh PB from 13 AML patients at the diagnosis. One-way ANOVA followed by Šidák's multiple comparisons test. g) Metabolic profile between AML male patients (n = 19, in blue) compared to female (n = 10, purple) in PB CD34$^{+}$ cells.

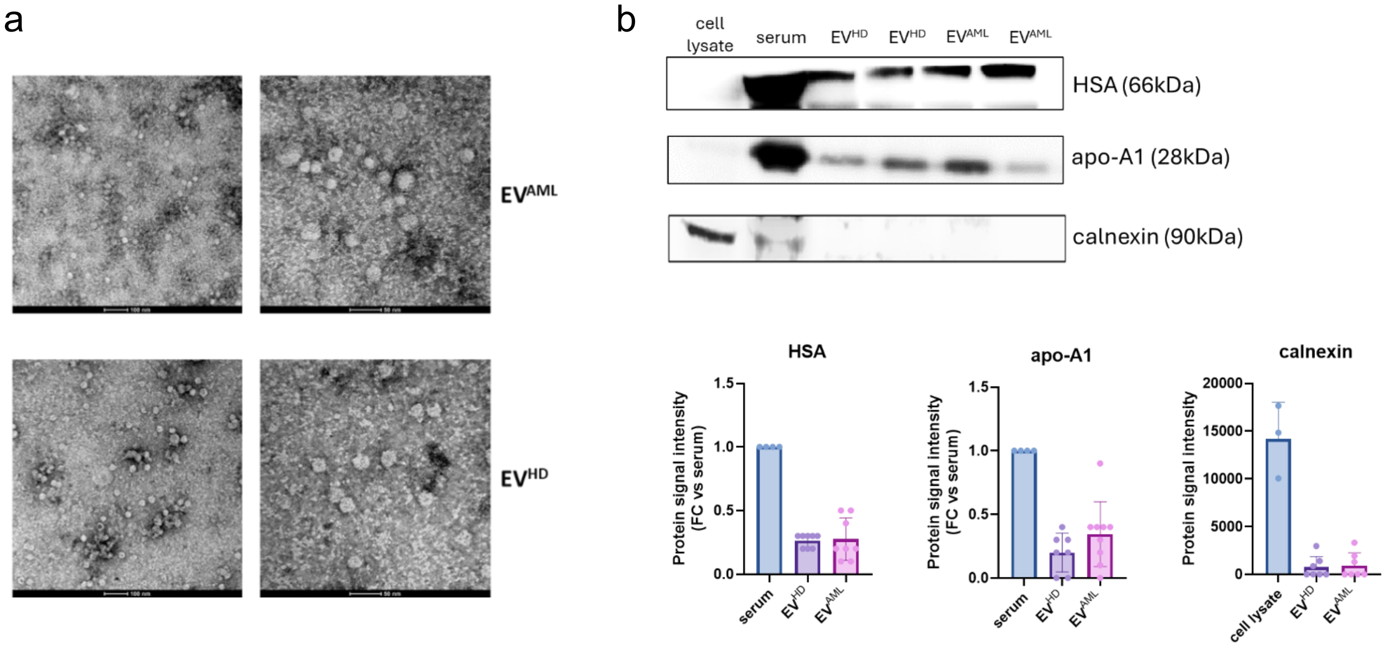


**Supplementary Figure 3**

Characterization of EVs from AML patients and HD. a) Representative image from Transmission electron microscopy (TEM) (n = 4); Scale bar 100nm (left) and 50 nm (right). b) Western blot analyses for contaminants. Human serum albumin (HSA; 66 kDa) and apo-A1 (28kDa) reduction in $EV^{HD}$ and $EV^{AML}$ compared to positive control with unprocessed AML serum. The absence of cell-specific marker calnexin (90kDa) in EV from HD and AML patients in comparison to the positive control with cell lysate. Densitometric analysis of western blot results presented was performed using image processing software. Data are presented as mean values ± SEM of three to four independent experiments.

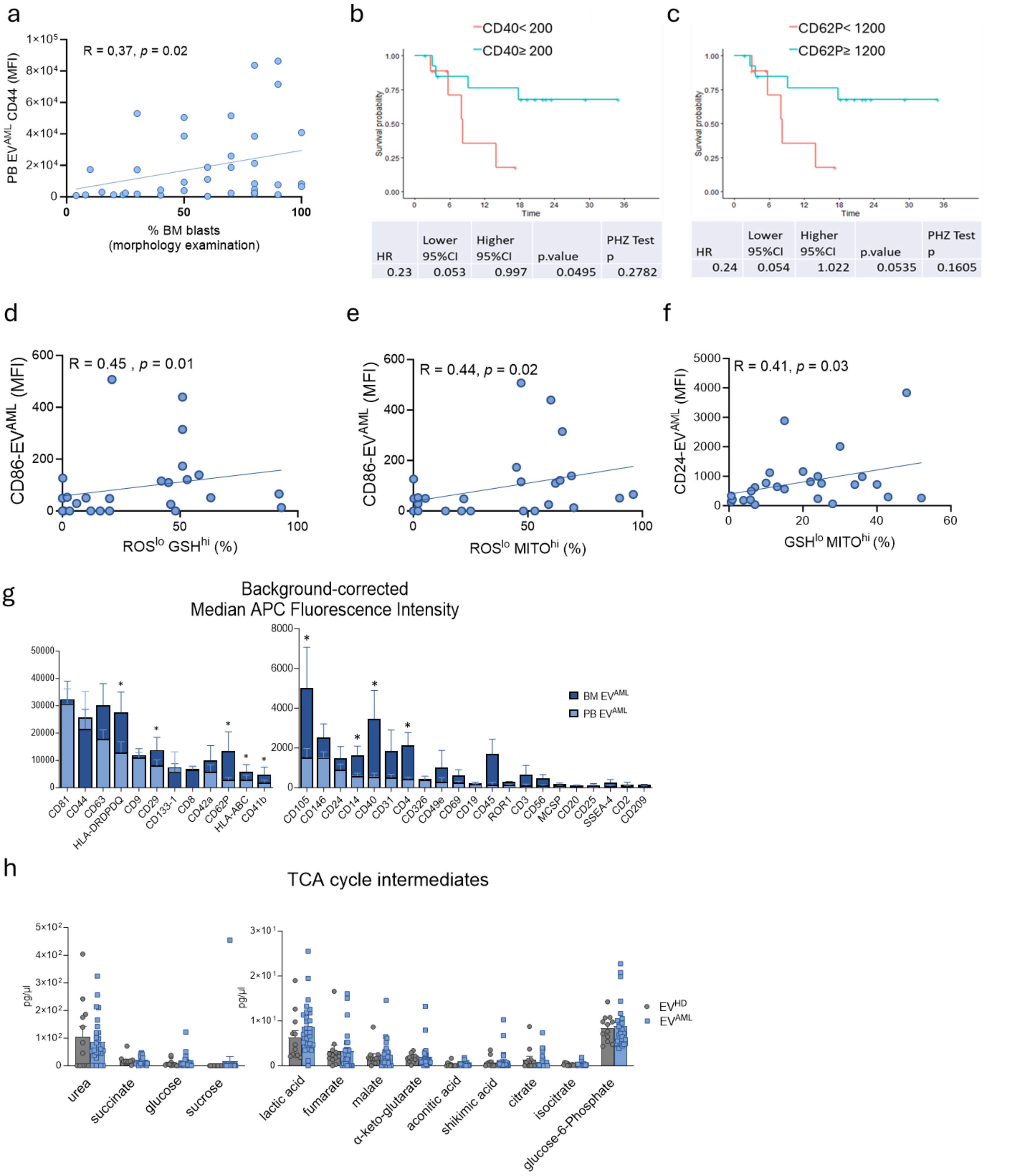


**Supplementary Figure 4**

a) Spearman correlation between CD44 expression on PB EV expressed as MFI and BM blast percentages detected by morphology examination (n=38, AML patients, R = 0.37, $P$ = 0.02). b-c) The Kaplan-Meier survival analyses based on the $EV^{AML}$ expression for CD40 and CD62P in AML patients who had not received chemotherapy (n = 23).

b) The OS rates of patients with low or high CD40 values (MFI cut-off = 200) expressed on $EV^{AML}$ for 36 months univariable Cox proportional-hazards model (HR = 0.23, 95% CI, 0.05−0.99, $p$ = 0.04). c) The OS rates of patients with low or high CD62P values (MFI cut-off = 1200) expressed on $EV^{AML}$ for 36 months univariable Cox proportional-hazards model (OS, HR = 0.24, 95% CI, 0.05−1.02, p = 0.05).d-e) Positive correlation between redox metabolic $CD34^{+}$ cell subsets $ROS^{lo}$ $MITO^{hi}$/$GSH^{hi}$ with CD86 MFI on $EV^{AML}$ (n = 27) (Spearman, R = 0.45/0.04, $p$ = 0.01/0.02, respectively). f) Positive correlation between redox metabolic $CD34^{+}$ cell subset $MITO^{hi}$ $GSH^{lo}$ with CD24 MFI on $EV^{AML}$ (n = 27) (Spearman, R= 0.41, $p$ = 0.03). g) Background-corrected median APC fluorescence intensity for 37 surface markers detected on $EV^{AML}$ from

PB (light blue) versus BM plasma (blue) of AML patients at diagnosis (n=10). Significance for HLA-DRDPDQ (*p* = 0,005), CD4 (*p* = 0,005), HLA-ABC (*p* = 0,007), CD40 (*p* = 0,01), CD41b (*p* = 0,027344), CD105 (*p* = 0,03), CD14 (*p* = 0,03), CD29 (*p* = 0,04), CD62P (*p* = 0,04) are reported using Multiple t-test with Wilcoxon matched-pairs signed rank test. h) Targeted metabolomic data on $EV^{AML}$ (n=27) versus $EV^{HD}$ (n=12). TCA cycle intermediates including urea, succinate, glucose, sucrose, lactic acid, fumarate, malate, α-keto-glutarate, aconitic acid, shikimic acid, citrate, isocitrate, glucose-6-phosphate expressed as pg/µl. No significant differences were reported between groups by two-way ANOVA.

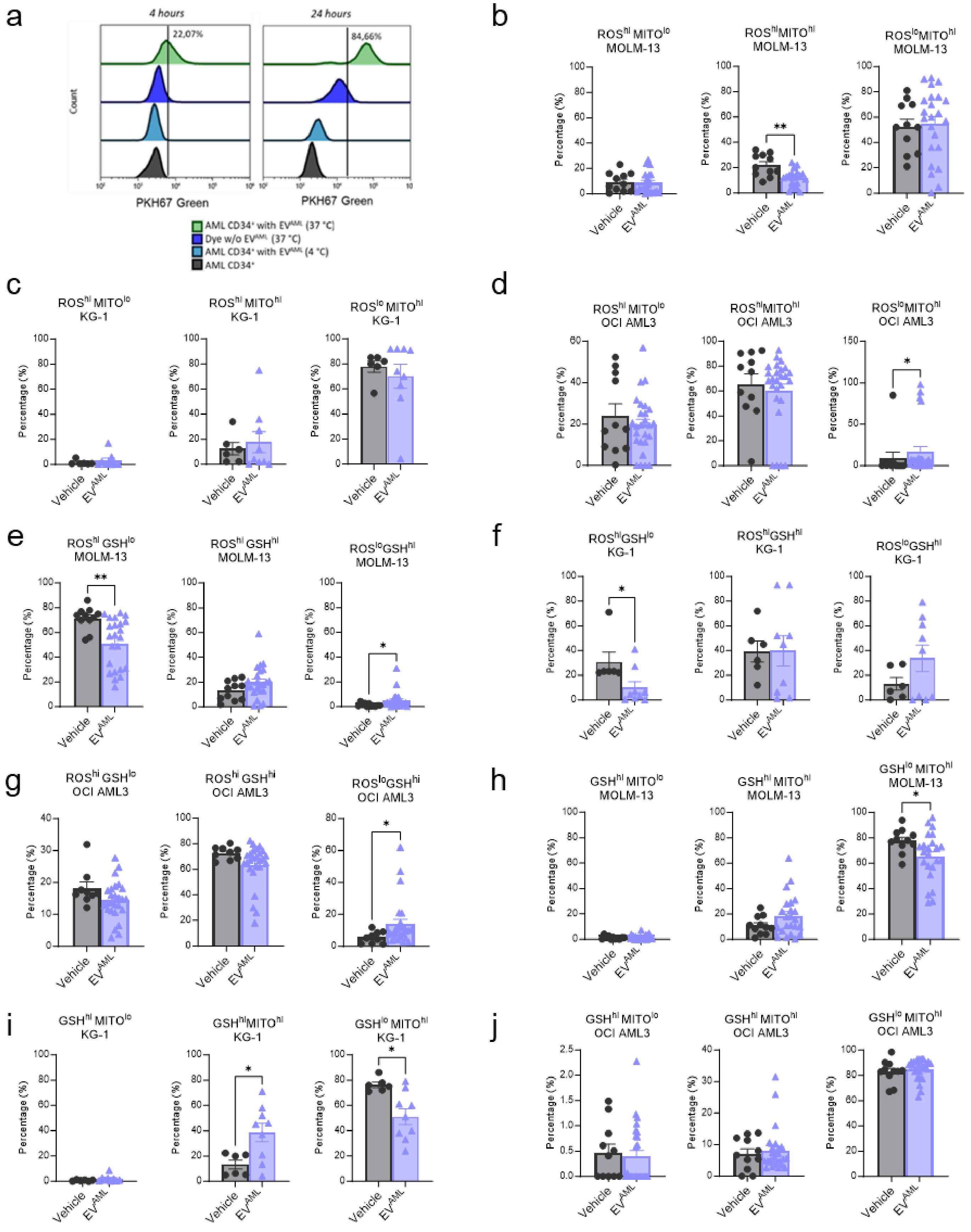


**Supplementary Figure 5** EV$^{AML}$ can be taken up by CD34$^+$ cells. a) Primary human CD34$^+$ AML cells were treated with PKH67-labeled EVs or with green dye PKH67 (without EVs) for 4 hours (left) or 24 hours (right) at 4°C and 37°C. Representative flow cytometry histograms showing the PKH67-labeled EV$^{AML}$ uptake by CD34$^+$ cells in the FITC channel (green histograms) in comparison to CD34$^+$ cells treated with PKH67 without EV$^{AML}$ (blue histograms) or with unstained EV$^{AML}$ (light blue histograms) or control CD34$^+$ cells (gray histograms). AML cell lines (e.g. MOLM-13) provided similar results. Data are presented as the mean values of 4 independent experiments.

Metabolic redox modulation by EVs of human leukemic cell lines from human leukemic cell lines including MOLM-13 (b, e, h), KG-1 (c, f, i) and OCI-AML3 (d, g, j).

b, c, d) ROS/MITO sub-fractions in leukemic cells treated for 24 hours with vehicle (PBS) or $EV^{AML}$: b, c, d) $ROS^{hi}$ $MITO^{lo}$, $ROS^{hi}$ $MITO^{hi}$, $ROS^{lo}$ $MITO^{hi}$;

e, f, g) ROS/GSH sub-fractions in leukemic cells treated for 24 hours with vehicle (PBS) or $EV^{AML}$: $ROS^{hi}$ $GSH^{lo}$, $ROS^{hi}$ $GSH^{hi}$, $ROS^{lo}$ $GSH^{hi}$.

h, i, j) GSH/MITO sub-fractions in leukemic cells treated for 24 hours with vehicle (PBS) or $EV^{AML}$: $GSH^{hi}$ $Mito^{lo}$, $GSH^{hi}$ $Mito^{hi}$, $GSH^{lo}$ $Mito^{hi}$. Significant differences were reported using Mann–Whitney test for unpaired samples. *p* values <0.05 (*), <0.01 (**) were considered significant.

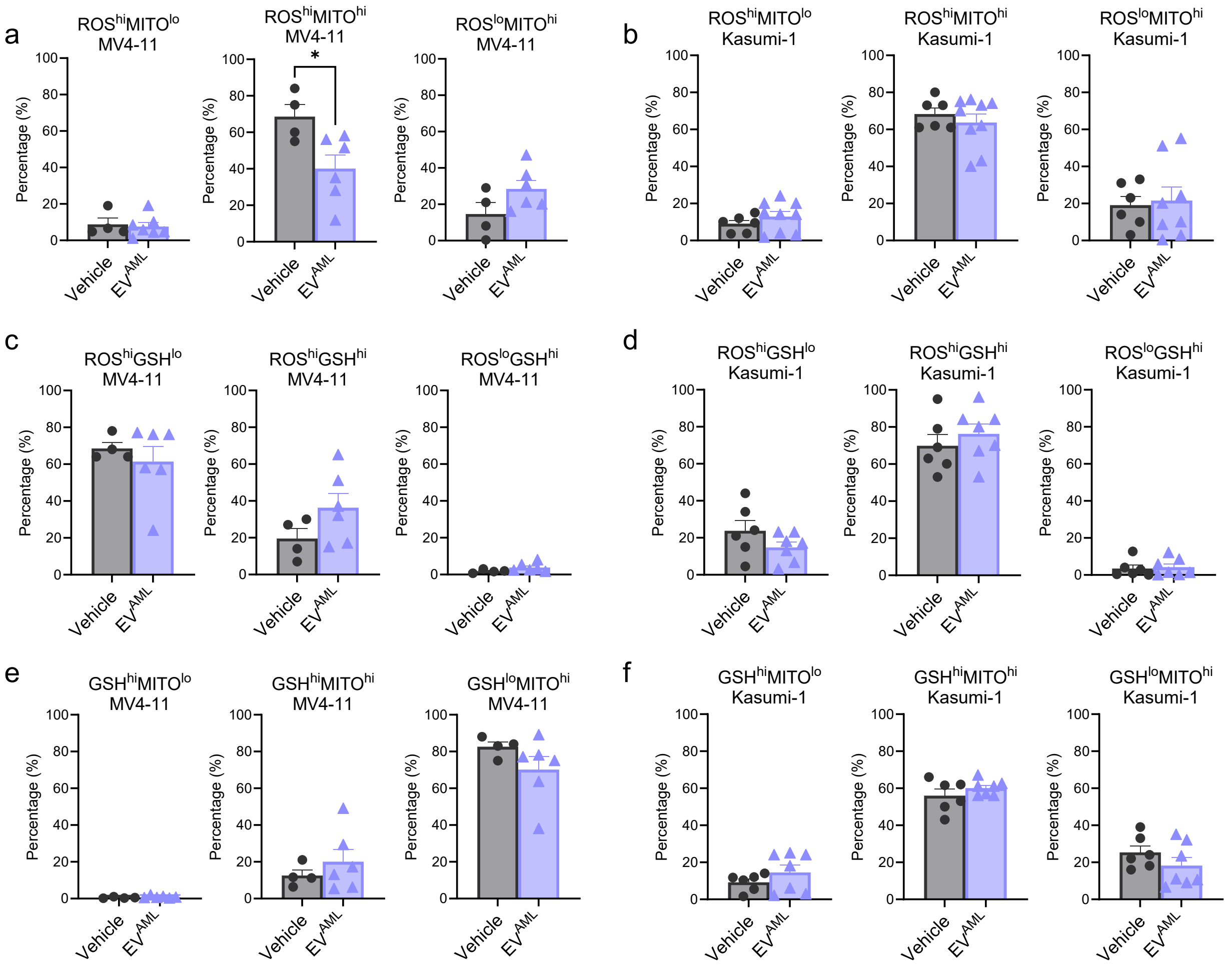


## Supplementary Figure 6

Metabolic redox modulation by EVs of human leukemic cell lines from human leukemic cell lines including MV4-11 and Kasumi-1 (n=4-6)/ n=6-7). a, b) ROS/MITO sub-fractions in leukemic cells treated for 24 hours with vehicle (PBS) or $EV^{AML}$: $ROS^{hi}$ $MITO^{lo}$, $ROS^{hi}$ $MITO^{hi}$, $ROS^{lo}$ $MITO^{hi}$ c, d) ROS/GSH sub-fractions in leukemic cells treated for 24 hours with vehicle (PBS) or $EV^{AML}$: $ROS^{hi}$ $GSH^{lo}$, $ROS^{hi}$ $GSH^{hi}$, $ROS^{lo}$ $GSH^{hi}$. e, f) GSH/MITO sub-fractions in leukemic cells treated for 24 hours with vehicle (PBS) or $EV^{AML}$: $GSH^{hi}$ $MITO^{lo}$, $GSH^{hi}$ $MITO^{hi}$, $GSH^{lo}$ $MITO^{hi}$. Significant differences were reported using Mann–Whitney test for unpaired samples. *p* values < 0.05 (*), < 0.01 (**) were considered significant.

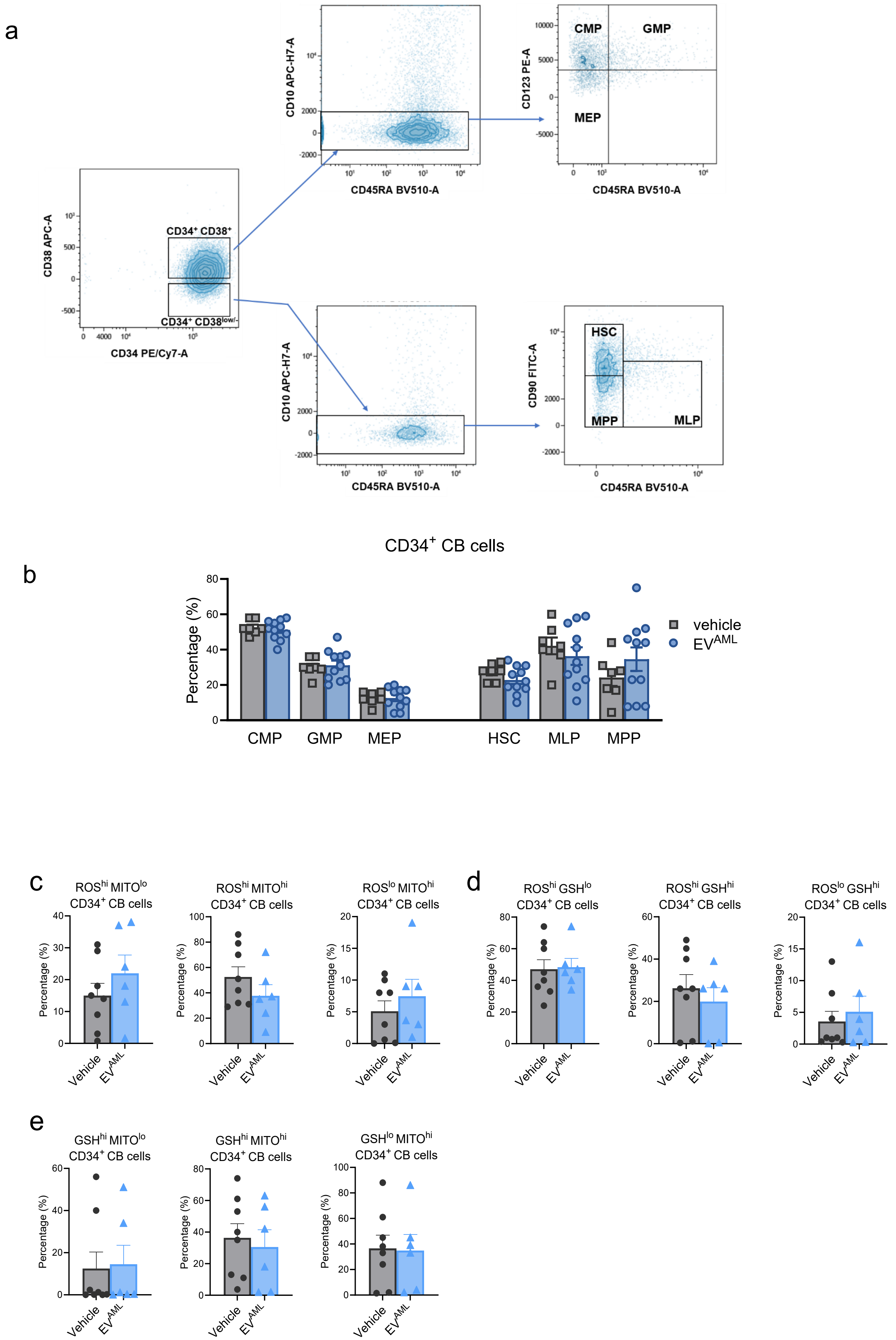
a
CD10 APC-H7-A
CD45RA BV510-A
CMP
GMP
MEP
CD123 PE-A
CD38 APC-A
CD34 PE/Cy7-A
CD34+ CD38+
CD34+ CD38low/-
HSC
MPP
MLP
CD90 FITC-A
b
CD34+ CB cells
Percentage (%)
vehicle
EVAML
CMP
GMP
MEP
HSC
MLP
MPP
c
ROShi MITOlo CD34+ CB cells
ROShi MITOhi CD34+ CB cells
ROSlo MITOhi CD34+ CB cells
d
ROShi GSHlo CD34+ CB cells
ROShi GSHhi CD34+ CB cells
ROSlo GSHhi CD34+ CB cells
e
GSHhi MITOlo CD34+ CB cells
GSHhi MITOhi CD34+ CB cells
GSHlo MITOhi CD34+ CB cells
Percentage (%)
Vehicle
EVAML

**Supplementary Figure 7**

a) Gating strategy 3. Gating strategy for identification of hematopoietic stem and progenitor cells on CB CD34$^+$ based on[1]. Common myeloid progenitor (CMP; CD123$^+$ CD45RA$^-$), megakaryocyte-erythroid progenitor (MEP; CD123$^-$ CD45RA$^-$) and granulocyte-macrophage progenitor (GMP; CD123$^+$ CD45RA$^+$) were gated from CD34$^+$ CD38$^+$/CD10$^-$ population. Hematopoietic stem cell (HSC; CD90$^+$ CD45RA) multipotent progenitor (MPP; CD45RA$^-$ CD90$^-$), lymphoid-primed multipotent progenitor (MLP; CD45RA$^+$ CD90$^-$) were gated from CD34$^+$ CD38$^{low/-}$ /CD10$^-$ population.

b) Frequency of hematopoietic stem/progenitor cell types including hematopoietic stem cell (HSC), common myeloid progenitor (CMP), multipotent progenitor (MPP), lymphoid-primed multipotent progenitor (MLP), granulocyte-macrophage progenitor (GMP), and megakaryocyte-erythroid progenitor (MEP) after treatments with vehicle (PBS) or EV$^{AML}$ by FACS analysis (n=8). No significant differences were reported using two-ANOVA.

Redox metabolic phenotype of CD34$^+$ isolated from AML patients in co-culture with vehicle (PBS) or EV$^{AML}$ for 24 hours and stained with ROS, MITO and GSH.

Metabolic redox modulation by EV$^{AML}$ on CD34$^+$ cells isolated from CB and treated with vehicle or EV$^{AML}$ for 24 hours. c) ROS/MITO sub-fractions in CB CD34$^+$ cells: ROS$^{hi}$ MITO$^{lo}$, ROS$^{hi}$ MITO$^{hi}$, ROS$^{lo}$ MITO$^{hi}$; d) ROS/GSH sub-fractions in CB CD34$^+$ cells: ROS$^{hi}$ GSH$^{lo}$, ROS$^{hi}$ GSH$^{hi}$, ROS$^{lo}$ GSH$^{hi}$. e) GSH/Mito sub-fractions in CB CD34$^+$ cells: GSH$^{hi}$ MITO$^{lo}$, GSH$^{hi}$ MITO$^{hi}$, GSH$^{lo}$ MITO$^{hi}$. No significant differences were reported using Mann–Whitney test for unpaired samples.

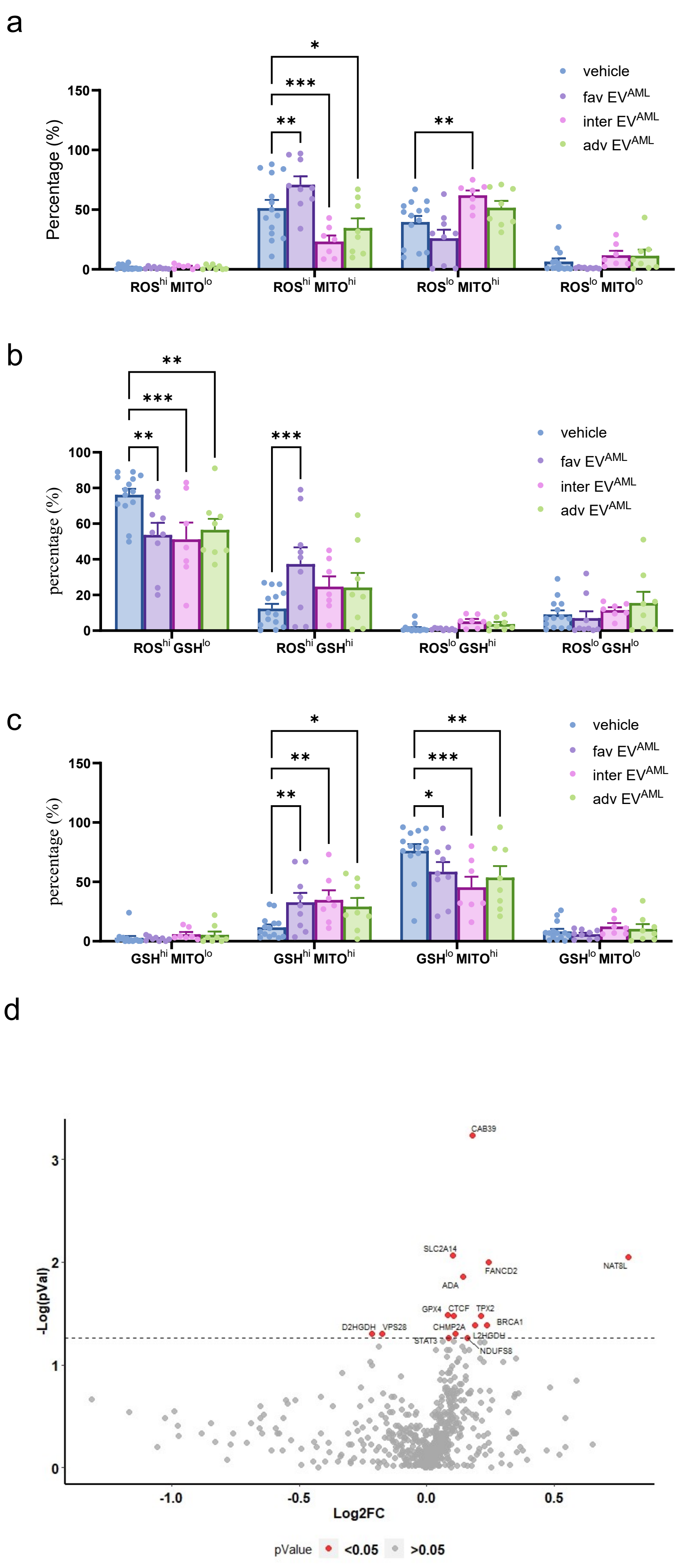


e

| GENE | *P* value |
|---|---|
| CAB39 | 0.00 |
| SLC2A14 | 0.01 |
| NAT8L | 0.01 |
| FANCD2 | 0.01 |
| ADA | 0.01 |
| GPX4 | 0.03 |
| CTCF | 0.03 |
| TPX2 | 0.03 |
| BRCA1 | 0.04 |
| L2HGDH | 0.04 |
| D2HGDH | 0.04 |
| VPS28 | 0.04 |
| CHMP2A | 0.04 |
| STAT3 | 0.04 |
| NDUFS8 | 0.04 |

f

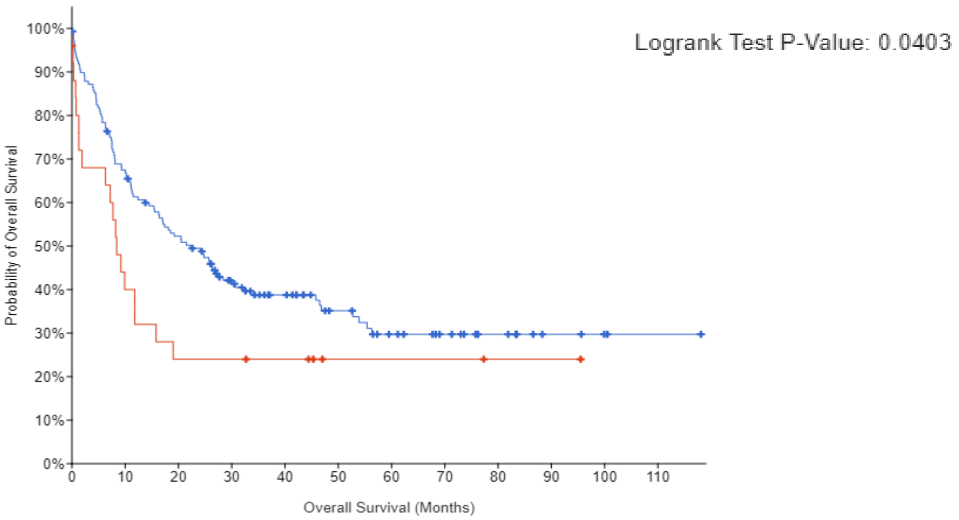

Logrank Test P-Value: 0.0403
Probability of Overall Survival
100%
90%
80%
70%
60%
50%
40%
30%
20%
10%
0%
0
10
20
30
40
50
60
70
80
90
100
110
Overall Survival (Months)


g

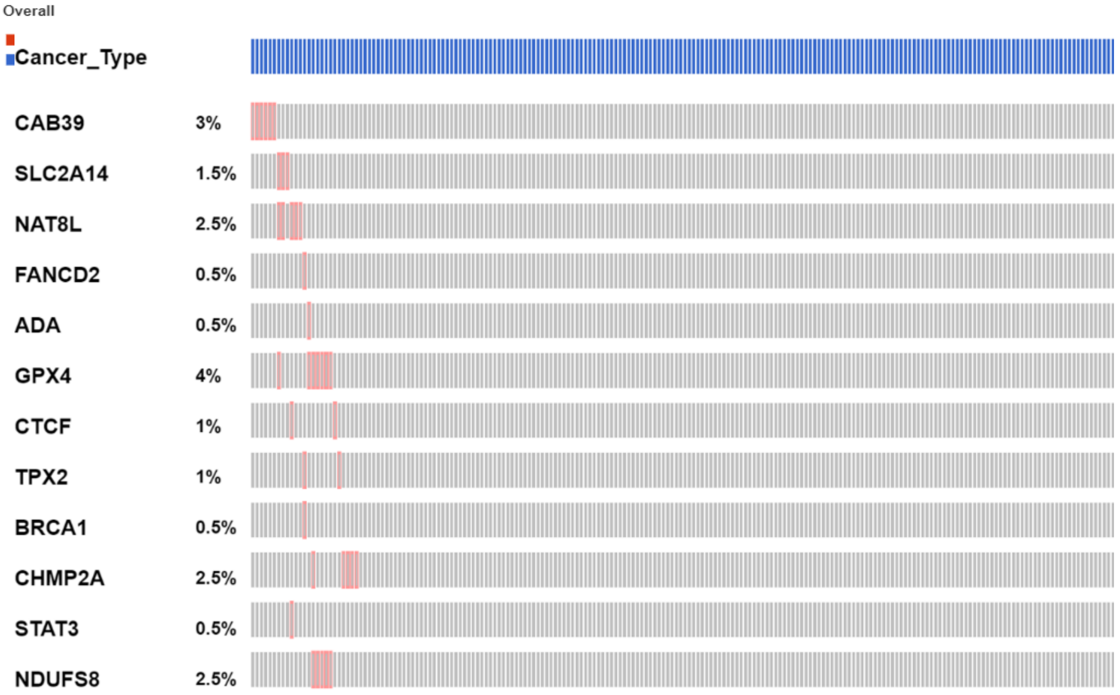

Overall
Cancer_Type
CAB39 3%
SLC2A14 1.5%
NAT8L 2.5%
FANCD2 0.5%
ADA 0.5%
GPX4 4%
CTCF 1%
TPX2 1%
BRCA1 0.5%
CHMP2A 2.5%
STAT3 0.5%
NDUFS8 2.5%


h

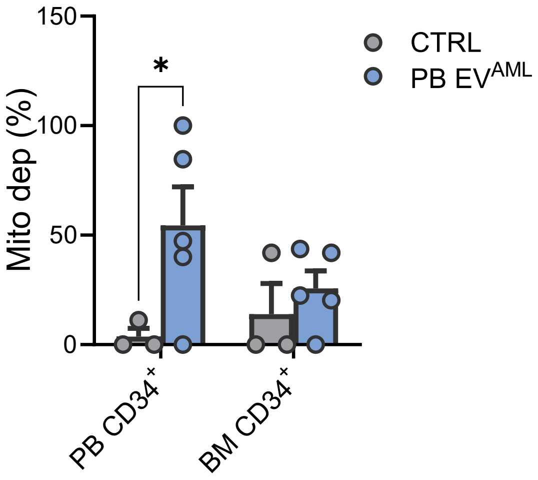

150
100
50
0
Mito dep (%)
*
CTRL
PB EV^AML
PB CD34+
BM CD34+


i

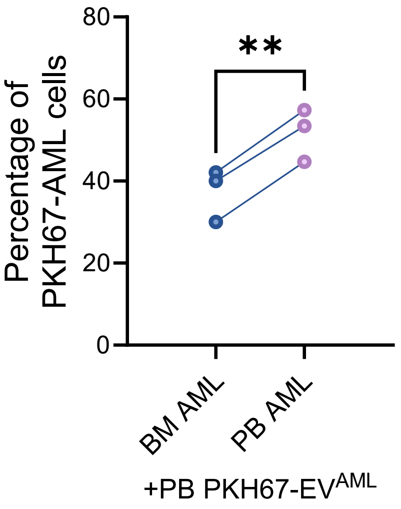

80
60
40
20
0
Percentage of PKH67-AML cells
**
BM AML
PB AML
+PB PKH67-EV^AML

**Supplementary Figure 8**

Redox metabolic profiling for $CD34^+$ AML cells treated for 24 hours with $EV^{AML}$ from adverse, intermediate or favorable-risk AML patients. ROS/MITO subsets expressed as percentages were reported for $CD34^+$ AML cells (a). Percentage of ROS/GSH subsets for CD34+ AML cells (b). Percentages of GSH/MITO subsets (c) for $CD34^+$ AML cells treated with favorable $EV^{AML}$ (n=9), intermediate $EV^{AML}$ (n=7) or adverse $EV^{AML}$ (n=8) from 10 independent experiments. Significant differences were reported by two-way ANOVA with Šidák's multiple comparisons test. NanoString nCounter™ profiling identifies pronounced gene expression changes in AML $CD34^+$ cells treated with vehicle (PBS) or $EV^{AML}$ for 24 hours. d) Volcano plot (fold change (log2) versus p value (log2)) depicting differential gene expression of 15 metabolic genes in cells (namely, *ADA, BRCA1, CAB39, CHMP2A, CTCF, D2HGDH, FANCD2, GPX4, L2HGDH, NAT8L, NDUFS8, SLC2A14, STAT3, TPX2, and VPS28*). Mann–Whitney test comparing EV-treated $CD34^+$ cells versus nontreated $CD34^+$ cells (vehicle) (*n* = 12-14). e) Differential genes expressed significantly in AML $CD34^+$ cells (namely *ADA, BRCA1, CAB39, CHMP2A, CTCF, D2HGDH, FANCD2, GPX4, L2HGDH, NAT8L, NDUFS8, SLC2A14, STAT3, TPX2, VPS28*) and their relative *p* value. f) The Kaplan-Meier overall survival curves of TCGA AML patients (n = 173 AML cases through cBioPortal: https://www.cbioportal.org/) grouped by specific metabolic-related genes up-regulated in our co-cultures system (*CAB39, ADA, SLC2A14, CHMP2A, FANCD2, NAT8L, GPX4, CTCF, TPX2, NDUFS8, STAT3, BRCA1*) with *p* value was calculated by log-rank ($p$ = 0.04). g) Oncoprint plot with the list of interrogated genes showing the percentage of AML patients with mRNA upregulation. h) Different metabolic differences between $CD34^+$ cells stained after treatment with $EV^{AML}$ or vehicle comparing PB versus paired BM fractions. (* $p < 0.05$) (n = 3). i) Differential uptake of stained PB EVAML with PKH67 on paired PB vs BM MNC derived from three AML patients at diagnosis after 24 hours in coculture. Statistical significances were reported by Mann–Whitney test for unpaired samples (** $p < 0.01$)

## Supplementary Tables

*Supplementary Table 1*

The clinicopathological features of all AML patients involved in the study

| **Characteristics** | | **N (%), median [range]** |
|---|---|---|
| **n** | | 114 |
| **Diagnosis (%)** | *de novo* | 81 (71%) |
| | secondary | 31 (27%) |
| | therapy-related | 2 (2%) |
| **WBC (x $10^9$/L)** | | 3.64 [0.73-340] |
| **Platelet count (x $10^9$/L)** | | 46 [8-296] |
| **Hemoglobin level (g/dl)** | | 9 [4.7-14.6] |
| **ELN 2022 risk group at diagnosis (%)** | favorable | 18 (16%) |
| | intermediate | 44 (38%) |
| | adverse | 52 (46%) |

*Supplementary Table 2*
Information on morphology examination (BM and PB blast counts) for all AML samples used for EV isolations and EV profiling

| | Morphology examination (%) | | | | | |
|---|---|---|---|---|---|---|
| UPN | BM blasts | PB blasts | diagnosis | ELN_2022 | WBC (x$10^9$/L) | EV profiling (MACSPlex) |
| ID_1 | 60 | 20 | novo | adv | 16,43 | Y |
| ID_2 | 24 | 13 | novo | adv | 1,19 | Y |
| ID_3 | 30 | 20 | novo | adv | 1,63 | Y |
| ID_4 | 70 | 88 | novo | adv | 18,58 | Y |
| ID_5 | N/A | 34 | novo | adv | 10,45 | Y |
| ID_6 | 90 | 90 | novo | adv | 25,47 | Y |
| ID_7 | 80 | 8,6 | novo | adv | 1,08 | Y |
| ID_8 | 30 | 7 | sec | adv | 19,72 | Y |
| ID_9 | 50 | 27 | ter | adv | 45,21 | Y |
| ID_10 | 60 | 3 | novo | adv | 1,67 | Y |
| ID_11 | 50 | 30 | novo | adv | 1,37 | Y |
| ID_12 | 80 | 35 | sec | adv | 1,82 | Y |
| ID_13 | 25 | 15 | sec | adv | 5,60 | Y |
| ID_14 | 80 | 51 | novo | int | 28,80 | Y |
| ID_15 | 80 | 12 | novo | int | 10,28 | Y |
| ID_16 | 70 | 15 | sec | int | 2,07 | Y |
| ID_17 | 100 | 66 | novo | int | 0,94 | Y |
| ID_18 | 80 | 8 | novo | int | 2,53 | Y |
| ID_19 | 80 | 67 | sec | int | 27,48 | Y |
| ID_20 | N/A | 68 | novo | int | 2,90 | Y |
| ID_21 | 20 | 2 | novo | int | 0,73 | Y |
| ID_22 | 70 | 46 | sec | int | 20,00 | Y |
| ID_23 | 100 | 95 | novo | int | 164,10 | Y |
| ID_24 | 50 | 0 | novo | int | 1,79 | Y |
| ID_25 | 40 | 0 | novo | int | 1,69 | Y |
| ID_26 | 80 | 89 | novo | int | 11,40 | Y |
| ID_27 | N/A | N/A | novo | fav | 7,20 | Y |
| ID_28 | 80 | 20 | novo | fav | 5,30 | Y |
| ID_29 | 100 | 66 | novo | fav | 115,57 | Y |
| ID_30 | 30 | 4 | novo | fav | 1,00 | Y |
| ID_31 | 90 | 87 | novo | int | 11,15 | Y |
| ID_32 | *4 | 2 | novo | int | 3,59 | Y |
| ID_33 | 60 | 2 | novo | int | 2,87 | Y |
| ID_34 | 90 | 30 | novo | adv | 10,09 | Y |
| ID_35 | 40 | 10 | novo | int | 0,91 | Y |
| ID_36 | *15 | N/A | novo | int | 2,05 | Y |
| ID_37 | 90 | 43 | novo | fav | 31,94 | Y |
| ID_38 | 70 | 44 | novo | adv | 7,68 | Y |
| ID_39 | *8 | 0 | novo | adv | 2,04 | Y |
| ID_40 | 10 | 1,5 | sec | fav | 111,05 | Y |
| ID_41 | 50 | 18 | novo | fav | N/A | Y |

** hemodiluted bone marrow aspirates or myeloid sarcoma (MS) or with specific AML-defining recurrent genetic abnormality; Abbreviations: N/A not available; sec: secondary AML; ter: Therapy-related AML; novo: de novo AML; Y: yes; adv/int/fav: adverse, intermediate and favorable ELN risk.*

*Supplementary Table 3*

Spearman correlation between EV surface markers and AML CD34$^+$ cells' metabolic status

| **Adverse-risk AML patients** | **Gluco dep (%)** |
|---|---|
| CD209 | R = -0.94 *p* = 0.0004 |
| CD142 | R = -0.84 *p* = 0.004 |
| CD31 | R = -0.83 *p* = 0.0083 |
| CD14 | R = -0.88 *p* = 0.003 |
| CD4 | R = -0.88 *p* = 0.003 |
| CD49e | R = -0.80 *p* = 0.01 |
| CD40 | R = -0.81 *p* = 0.01 |
| CD44 | R = -0.80 *p* = 0.01 |
| CD133-1 | R = -0.78 *p* = 0.01 |

| **Intermediate-risk AML patients** | **Gluco dep (%)** |
|---|---|
| CD209 | R = 0.66 *p* = 0.04 |
| CD14 | R = 0.66 *p* = 0.03 |
| MCSP | R = 0.61 *p* = 0.04 |
| SSEA-4 | R = 0.66 *p* = 0.03 |

*Supplementary Table 4*
Lipidomic datasets divided for lipid classes for both the $EV^{AML}$ (*n* = 16) and $EV^{HD}$ samples (*n* = 17). Significant differences were reported between $EV^{HD}$ and EVAML using the Mann-Whitney unpaired t-test.

| ***Lipid subclass: abbreviations, full name*** | | ***Annotated molecular species, n*** | ***$EV^{AML}$ nMol/mL*** | ***AML SEM*** | ***$EV^{HD}$ nMol/mL*** | ***HD SEM*** | ***P*** |
|---|---|---|---|---|---|---|---|
| Cer | Ceramide | 15 | 269.09 | 29.40 | 261.78 | 28.73 | 0.42 |
| DG | Diacylglycerol | 67 | 324.94 | 40.57 | 218.80 | 20.45 | 0.01* |
| FA | Fatty acid | 20 | 400.58 | 22.94 | 340.28 | 21.51 | 0.03* |
| LPC | Lysophophatidylcholine | 30 | 60.08 | 10.33 | 50.93 | 5.57 | 0.21 |
| LPE | Lysophosphatidyl ethanolamine | 2 | 1.17 | 0.35 | 0.80 | 0.11 | 0.15 |
| PC | Phosphatidylcholine | 51 | 994.11 | 79.53 | 1090.01 | 91.95 | 0.21 |
| PE | Phosphatidylethanolamine | 19 | 61.58 | 7.13 | 47.30 | 6.47 | 0.07 |
| PI | Phosphatidylinositol | 8 | 20.54 | 2.20 | 23.92 | 2.61 | 0.16 |
| SM | Sphingomyelin | 55 | 239.80 | 15.92 | 255.63 | 11.48 | 0.20 |

*Supplementary Table 5*
MRM transitions for GC-QQQ-MS analysis. RT= Retention time; CE = Collision Energy.

| **Compound** | **RT (min)** | **Quantifier** | **CE** | **Qualifier** | **CE** |
|---|---|---|---|---|---|
| aconitic acid | 5.24 | 375→147 | 10 | 375→211 | 10 |
| alpha-keto-glutaric acid | 4.75 | 288→73 | 20 | 288→198 | 10 |
| alpha-ketoglutaric acid (IS) | 4.75 | 308→147 | 10 | - | - |
| citric acid | 5.42 | 183→73 | 10 | 183→183 | 10 |
| citric acid (IS) | 5.42 | 276→185 | 10 | - | - |
| fumaric acid | 4.00 | 245→73 | 20 | 245→245 | 10 |
| fumaric acid (IS) | 4.00 | 249→147 | 10 | - | - |
| glucose | 5.7 | 319→129 | 10 | 319→157 | 10 |
| glucose (IS) | 5.7 | 323→132 | 10 | - | - |
| glucose-6-phosphate | 6.7 | 387→387 | 10 | 387→73 | 10 |
| isocitric acid | 5.47 | 245→73 | 20 | 245→83 | 20 |
| lactic acid | 2.72 | 219→147 | 10 | 219→191 | 10 |
| malic acid | 4.5 | 335→147 | 10 | 335→73 | 10 |
| malic acid (IS) | 4.5 | 339→147 | 20 | - | - |
| methyl stearate (IS) | 6.2 | 298→101 | 20 | - | - |
| shikimic acid | 5.4 | 462→204 | 10 | 462→254 | 20 |
| succinic acid | 3.9 | 262→73 | 10 | 262→113 | 10 |
| succinic acid (IS) | 3.9 | 251→147 | 20 | - | - |
| sucrose | 7.31 | 437→257 | 20 | 437→303 | 10 |
| sucrose (IS) | 7.31 | 442→262 | 10 | - | - |
| urea | 3.35 | 261→147 | 20 | 261→245 | 10 |

## Supplementary References

1. Trino, S. *et al.* Acute Myeloid Leukemia Cells Functionally Compromise Hematopoietic Stem/Progenitor Cells Inhibiting Normal Hematopoiesis Through the Release of Extracellular Vesicles. *Front Oncol* **12**, 824562 (2022).
