# Supplementary material for: Parallel single-cell metabolic analysis and extracellular vesicle profiling reveal vulnerabilities with prognostic significance in acute myeloid leukemia
Source: Nat Commun. 2024 Dec 30;15:10878. doi: 10.1038/s41467-024-55231-9 (PMC11685939; doi:10.1038/s41467-024-55231-9)
Supplement: Supplementary file 2 — Reporting Summary [file 41467_2024_55231_MOESM2_ESM.pdf]

nature portfolio

Corresponding author(s): Dr. Antonio Curti

Last updated by author(s): Nov 18, 2024

Reporting Summary

Nature Portfolio wishes to improve the reproducibility of the work that we publish. This form provides structure for consistency and transparency in reporting. For further information on Nature Portfolio policies, see our Editorial Policies and the Editorial Policy Checklist.

Statistics

For all statistical analyses, confirm that the following items are present in the figure legend, table legend, main text, or Methods section.

n/a	Confirmed	
☐	☒	The exact sample size (*n*) for each experimental group/condition, given as a discrete number and unit of measurement
☐	☒	A statement on whether measurements were taken from distinct samples or whether the same sample was measured repeatedly
☐	☒	The statistical test(s) used AND whether they are one- or two-sided<br>*Only common tests should be described solely by name; describe more complex techniques in the Methods section.*
☐	☒	A description of all covariates tested
☐	☒	A description of any assumptions or corrections, such as tests of normality and adjustment for multiple comparisons
☐	☒	A full description of the statistical parameters including central tendency (e.g. means) or other basic estimates (e.g. regression coefficient) AND variation (e.g. standard deviation) or associated estimates of uncertainty (e.g. confidence intervals)
☐	☒	For null hypothesis testing, the test statistic (e.g. *F*, *t*, *r*) with confidence intervals, effect sizes, degrees of freedom and *P* value noted<br>*Give P values as exact values whenever suitable.*
☒	☐	For Bayesian analysis, information on the choice of priors and Markov chain Monte Carlo settings
☒	☐	For hierarchical and complex designs, identification of the appropriate level for tests and full reporting of outcomes
☐	☒	Estimates of effect sizes (e.g. Cohen's *d*, Pearson's *r*), indicating how they were calculated

Our web collection on statistics for biologists contains articles on many of the points above.

Software and code

Policy information about availability of computer code

Data collection	Seahorse XFp Analyzer, inverted microscope (Axiovert 40, Zeiss), BD FACSAria™ Fusion Special Order (SORP) cell sorter cytometer (BD Biosciences), Sony ID7000™ Spectral Cell Analyzer, NanoSight LM10 system (NanoSight Ltd.), CytoFLEX V5-B5-R3 (Beckman Coulter), 5L Cytek Aurora spectral cytometer, 1260 Infinity II LC System coupled with an Agilent 6530 Q-TOF spectrometer (Agilent Technologies), Tecnai G2 transmission electron microscope (FEI Company), ChemiDoc XRS+ System (Bio-Rad), Veleta digital camera (Olympus Soft Imaging System), Gas chromatography (GC)-triple quadrupole Mass Spectrometry GC-QQQ-MS,
Data analysis	Data were analyzed using the following programs/softwares and were described in the relevant section of Methods:<br>Kaluza Analysis 2.1<br>GraphPad prism 9.3.1/10<br>Microsoft Excel 2019<br>Agilent Seahorse Wave<br>CytExpert<br>R Software v4.0.4<br>nSolver Analysis Software 4.0<br>NTA 2.3 analytic software<br>MS-DIAL software<br>ImageJ<br>LipidOne<br>MetaboAnalist 5.0 web platform<br>Living Image Software (version 4.7.2, PerkinElmer)

BioPAN
ImageJ software

For manuscripts utilizing custom algorithms or software that are central to the research but not yet described in published literature, software must be made available to editors and reviewers. We strongly encourage code deposition in a community repository (e.g. GitHub). See the Nature Portfolio guidelines for submitting code & software for further information.

Data

Policy information about availability of data

All manuscripts must include a data availability statement. This statement should provide the following information, where applicable:

- Accession codes, unique identifiers, or web links for publicly available datasets
- A description of any restrictions on data availability
- For clinical datasets or third party data, please ensure that the statement adheres to our policy

The publicly available datasets used in this study can be accessed at cBioPortal for Cancer Genomics: CAB39, ADA and 10 other genes in Acute Myeloid Leukemia (TCGA, NEJM 2013). The gene expression profile data generated in this study are available in the Gene Expression Omnibus (GEO) repository at GEO Accession viewer (https://www.ncbi.nlm.nih.gov/geo/query/acc.cgi?acc=GSE245810) under accession number GSE245810. All relevant experimental data regarding the isolation protocol and general characterization have been submitted to the EV-TRACK knowledgebase (EV-TRACK ID: EV230004) EV-TRACK (https://evtrack.org/search_results.php?evtraqid=EV230004&submit=1). Lipidomic and metabolomic data on EVs have been deposited to the EMBL-EBI MetaboLights database (DOI: 10.1093/nar/gkad1045, PMID:37971328) with the identifier MTBLS11523 for lipidomics (https://www.ebi.ac.uk/metabolights/MTBLS11523) and MTBLS11746 for metabolomics (https://www.ebi.ac.uk/metabolights/MTBLS11746). The protein surface data on EVs generated in this study are available in Zenodo (Zenodo) under accession code 10.5281/zenodo.14017145. Source data are provided with this paper.
Additional information is available upon request to the corresponding author: Dr. Antonio Curti, antonio.curti2@unibo.it

Research involving human participants, their data, or biological material

Policy information about studies with human participants or human data. See also policy information about sex, gender (identity/presentation), and sexual orientation and race, ethnicity and racism.

Reporting on sex and gender	We considered sex as one cofactor of interest in our work. Sex was assigned based on medical records after signing the informed consent form. However, we do not consider gender in our study design.
Reporting on race, ethnicity, or other socially relevant groupings	No reports were collected on race, ethnicity, or other socially relevant groupings
Population characteristics	Besides the sex dimension, the main covariate characteristics that were considered for this study were the European LeukemiaNet (ELN) 2022 categories (favorable, intermediate and adverse) and the therapy type (conventional chemotherapy versus no chemotherapy). The clinical findings of the AML patients are summarized in Supplementary Table 1 and better detailed in each corresponding figure legends.
Recruitment	All newly diagnosed AML patients were recruited at IRCCS Azienda Ospedaliero-Universitaria, Seràgnoli Hematology Institute in Bologna.
Ethics oversight	Clinical samples and data were collected after written informed consent. The research was approved by the institutional review board of the Area Vasta Emilia Centro (AVEC) Ethical Committee (approval code: 94/2016/O/Tess).

Note that full information on the approval of the study protocol must also be provided in the manuscript.

Field-specific reporting

Please select the one below that is the best fit for your research. If you are not sure, read the appropriate sections before making your selection.

☒ Life sciences ☐ Behavioural & social sciences ☐ Ecological, evolutionary & environmental sciences

For a reference copy of the document with all sections, see nature.com/documents/nr-reporting-summary-flat.pdf

Life sciences study design

All studies must disclose on these points even when the disclosure is negative.

Sample size	According to normal clinical practice, both peripheral blood samples (n = 114) and paired bone marrow aspirates (n=31) were collected from AML patients at diagnosis. PB samples were also collected from sex/age-matched Healthy Donors upon signed informed consent (n = 30). No power calculation was performed and we included all the patients as possible enrolled at the diagnosis. The sample size used in each experiment was not predetermined or formally justified for statistical power. However, sample size was determined based on availability of clinical sample in line to those reported in other publications with primary samples.
Data exclusions	No data were excluded.
Replication	Experiments that could not be reproduced were not included in this manuscript. Every experiments performed for the study were a result of a

	replication and the number of replicates with exact numbers are provided in the corresponding figure legend. At least three biological replicates were analyzed per group.
Randomization	In general, there was no randomization since the groups tested were defined by their characteristics (e.g. ELN risks). However, in the manuscript we reported when covariates as age, sex or therapy type were controlled. The selection of cells or extracellular vesicles from patients for each in vitro experiments has been done randomly.
Blinding	Blinding was not required in this study. However, researchers were blinded to group allocations for data generations of gene expression and lipidomic/metabolomic profiling.

Behavioural & social sciences study design

All studies must disclose on these points even when the disclosure is negative.

Study description	*Briefly describe the study type including whether data are quantitative, qualitative, or mixed-methods (e.g. qualitative cross-sectional, quantitative experimental, mixed-methods case study).*
Research sample	*State the research sample (e.g. Harvard university undergraduates, villagers in rural India) and provide relevant demographic information (e.g. age, sex) and indicate whether the sample is representative. Provide a rationale for the study sample chosen. For studies involving existing datasets, please describe the dataset and source.*
Sampling strategy	*Describe the sampling procedure (e.g. random, snowball, stratified, convenience). Describe the statistical methods that were used to predetermine sample size OR if no sample-size calculation was performed, describe how sample sizes were chosen and provide a rationale for why these sample sizes are sufficient. For qualitative data, please indicate whether data saturation was considered, and what criteria were used to decide that no further sampling was needed.*
Data collection	*Provide details about the data collection procedure, including the instruments or devices used to record the data (e.g. pen and paper, computer, eye tracker, video or audio equipment) whether anyone was present besides the participant(s) and the researcher, and whether the researcher was blind to experimental condition and/or the study hypothesis during data collection.*
Timing	*Indicate the start and stop dates of data collection. If there is a gap between collection periods, state the dates for each sample cohort.*
Data exclusions	*If no data were excluded from the analyses, state so OR if data were excluded, provide the exact number of exclusions and the rationale behind them, indicating whether exclusion criteria were pre-established.*
Non-participation	*State how many participants dropped out/declined participation and the reason(s) given OR provide response rate OR state that no participants dropped out/declined participation.*
Randomization	*If participants were not allocated into experimental groups, state so OR describe how participants were allocated to groups, and if allocation was not random, describe how covariates were controlled.*

Ecological, evolutionary & environmental sciences study design

All studies must disclose on these points even when the disclosure is negative.

Study description	*Briefly describe the study. For quantitative data include treatment factors and interactions, design structure (e.g. factorial, nested, hierarchical), nature and number of experimental units and replicates.*
Research sample	*Describe the research sample (e.g. a group of tagged Passer domesticus, all Stenocereus thurberi within Organ Pipe Cactus National Monument), and provide a rationale for the sample choice. When relevant, describe the organism taxa, source, sex, age range and any manipulations. State what population the sample is meant to represent when applicable. For studies involving existing datasets, describe the data and its source.*
Sampling strategy	*Note the sampling procedure. Describe the statistical methods that were used to predetermine sample size OR if no sample-size calculation was performed, describe how sample sizes were chosen and provide a rationale for why these sample sizes are sufficient.*
Data collection	*Describe the data collection procedure, including who recorded the data and how.*
Timing and spatial scale	*Indicate the start and stop dates of data collection, noting the frequency and periodicity of sampling and providing a rationale for these choices. If there is a gap between collection periods, state the dates for each sample cohort. Specify the spatial scale from which the data are taken*
Data exclusions	*If no data were excluded from the analyses, state so OR if data were excluded, describe the exclusions and the rationale behind them, indicating whether exclusion criteria were pre-established.*
Reproducibility	*Describe the measures taken to verify the reproducibility of experimental findings. For each experiment, note whether any attempts to repeat the experiment failed OR state that all attempts to repeat the experiment were successful.*

Randomization

Describe how samples/organisms/participants were allocated into groups. If allocation was not random, describe how covariates were controlled. If this is not relevant to your study, explain why.

Blinding

Describe the extent of blinding used during data acquisition and analysis. If blinding was not possible, describe why OR explain why blinding was not relevant to your study.

Did the study involve field work? ☐ Yes ☐ No

Field work, collection and transport

Field conditions

Describe the study conditions for field work, providing relevant parameters (e.g. temperature, rainfall).

Location

State the location of the sampling or experiment, providing relevant parameters (e.g. latitude and longitude, elevation, water depth).

Access & import/export

Describe the efforts you have made to access habitats and to collect and import/export your samples in a responsible manner and in compliance with local, national and international laws, noting any permits that were obtained (give the name of the issuing authority, the date of issue, and any identifying information).

Disturbance

Describe any disturbance caused by the study and how it was minimized.

Reporting for specific materials, systems and methods

We require information from authors about some types of materials, experimental systems and methods used in many studies. Here, indicate whether each material, system or method listed is relevant to your study. If you are not sure if a list item applies to your research, read the appropriate section before selecting a response.

Materials & experimental systems

n/a	Involved in the study	
☐	☒	Antibodies
☐	☒	Eukaryotic cell lines
☒	☐	Palaeontology and archaeology
☐	☒	Animals and other organisms
☐	☒	Clinical data
☒	☐	Dual use research of concern
☒	☐	Plants

Methods

n/a	Involved in the study	
☒	☐	ChIP-seq
☐	☒	Flow cytometry
☒	☐	MRI-based neuroimaging

Antibodies

Antibodies used

The following antibodies were used for flow cytometry:
CD45-PE (clone: HI30; BD Biosciences),
CD34-APC (clone 8G12; BD Biosciences),
CD38-PE/cyanine7 (clone HIT2; BioLegend) or CD38 APC (clone: HIT2; Biolegend)
CD3-Alexa Fluor 700 (clone SK7; BioLegend)
anti-puromycin monoclonal antibody AF647 or AF488 provided by R. Argüello (CIML) (clone: R4743L-E8, http://www.scenith.com)
CD34-APC (clone 8G12; BD Biosciences) or CD34 Pe/Cy7 (clone: 4H11; eBioscience™)
CD33-BV650 (clone: WM53, Sony Biotechnology)
CD117-BV605 (clone: 104D2, Sony Biotechnology)
CD45-BV750 (clone: HI30, Sony Biotechnology)
HLA-DR APC/H7 (clone: L243, BDTM)
CD123 PE (clone: 9F5; BD™) or CD123 PE (clone: 9F5; BD™)
CD90 FITC (clone: 5E10; BD Pharmingen™)
CD45RA-BV510 (clone: HI100; Biolegend)
CD10 APC-H7 (clone HI10a; BD™). Cells were incubated with antibodies at a 1:100 dilution, or as specified otherwise.
In selected experiments for spectral flow-cytometry, we used the following antibodies: CD34-BUV395 (clone: 563; BD Biosciences), CD33-BUV563 (clone: WM53; BD Biosciences), CD45-BUV805 (clone: HI30; BD Biosciences), CD38-BV711 (clone: HIT2; Biolegend), CD117 cFluor BYG667 (clone: 104D2; Cytek Biosciences), HLA-DR-PE-Fire 810 (clone: L243; Biolegend), and CD123-cFluor R720 (clone: 6H6; Cytek Biosciences), at a dilution of 1:50. We also used several dyes, namely CellROX Green for ROS levels (ROS: 0.8 μM), MitoTracker™ Red CMXRos for mitochondria potential/functionality (MITO: 50 nm), ThiolTracker™ Violet as glutathione detection reagent (GSH: 16 μM) and eBioscience™ Fixable Viability Dye eFluor™ 780 for cell viability according to the manufacturer's instructions (all from Thermo Fisher Scientific, Waltham, MA, USA).

The following primary antibodies were used for western blot analysis on EVs:

	mouse anti-ADP-ribosylation factor 6 (ARF6; clone: 3A-1, 1:1000; Santa Cruz Biotechnology), mouse anti-tumor susceptibility gene 101 (TSG101; clone: C-2, 1:1000; Santa Cruz Biotechnology), mouse anti-CD81 (clone: B-11, 1:1000; Santa Cruz Biotechnology), mouse anti-apoA-I (clone: B-10, 1:1000; Santa Cruz Biotechnology, Dallas, Texas, USA), rabbit anti-flotillin (clone: EPR6041, 1:1000; Abcam), rabbit anti-Calnexin antibody (clone: EPR3633(2), 1:1000; Abcam), rabbit anti-HSA (MA5-29022, 1:1000; Invitrogen) and rabbit anti-glutathione peroxidase GPX3 (PA5-119141, 1:500; Thermo Fischer Scientific, Waltham). Horseradish peroxidase (HRP)-conjugated anti-rabbit immunoglobulin G (1:10000; GE Healthcare, Amersham, UK) or anti-mouse IgG (1:10000; GE Healthcare) were used as secondary antibodies.<br>The PKH67 Green fluorescent cell linker (Sigma–Aldrich, Milan, Italy) was used to label the EV membrane. The EV from AML patients were labeled with the dye (1:80) for 5 min in Diluent C. MACSPlex analysis was performed on EVs using the MACSPlex Exosome Kit, human (Miltenyi Biotec, Bergisch Gladbach, Germany), which detects 37 exosomal surface epitopes plus two isotype controls, according to manufacturer's instructions. Briefly, after overnight incubation at RT of isolated EVs diluted in MACSPlex buffer with MACSPlex exosome capture beads, MACSPlex Exosome Detection Reagent for CD9, CD63, and CD81 (15 µl) was added to each tube with MACSPlex buffer (135 µl), followed by incubation for 1 hour at RT.
Validation	Antibodies were all purchased and validated by the suppliers. Validation statement for each antibody or dye listed above can be found on the manufacture's website. For FACS staining, antibodies were validated in our lab using appropriate negative controls and right concentrations after titration.

Eukaryotic cell lines

Policy information about cell lines and Sex and Gender in Research

Cell line source(s)	The human AML cell lines MOLM-13 (#ACC 554), OCI-AML3 (#ACC 582), KG-1 (#ACC 14), Kasumi-1 (#ACC 220) and MV4-11 (# ACC 102) were purchased from DSMZ (Braunschweig, Germany)
Authentication	Authetications were performed by manufacturer by STR analysis according to the global standard ANSI/ATCC ASN-0002.1-2021 (2021). Cell morphology was checked regularly.
Mycoplasma contamination	All cell lines were tested negative for mycoplasma contamination
Commonly misidentified lines (See ICLAC register)	none

Animals and other research organisms

Policy information about studies involving animals; ARRIVE guidelines recommended for reporting animal research, and Sex and Gender in Research

Laboratory animals	6–8-week-old male NOD-scid IL2R gamma null (NSG) mice (n=5/group; two independent experiments) at point of transplant
Wild animals	None
Reporting on sex	No sex-based analyses performed. In this study, given the higher incidence of AML and better engraftment efficiency observed in male cohorts, male mice were prioritized to ensure consistency across experiments.
Field-collected samples	None
Ethics oversight	Experiments involving animals were approved by the Italian Ministry of Health and have been done in accordance with the applicable Italian laws (D.L.vo 26/14 and following amendments), the Institutional Animal Care and Use Committee, and the institutional guidelines at the European Institute of Oncology.

Note that full information on the approval of the study protocol must also be provided in the manuscript.

Clinical data

Policy information about clinical studies

All manuscripts should comply with the ICMJEguidelines for publication of clinical research and a completedCONSORT checklist must be included with all submissions.

Clinical trial registration	Not available
Study protocol	The research was approved by the institutional review board of the Area Vasta Emilia Centro (AVEC) Ethical Committee (approval code: 94/2016/O/Tess).
Data collection	Clinical data were collected after obtaining written informed consent
Outcomes	Time-to-event overall survival (OS) and progression-free survival (PFS) endpoints were assessed following the Kaplan–Meier methods, and relative survival was provided.

Plants

Seed stocks

Report on the source of all seed stocks or other plant material used. If applicable, state the seed stock centre and catalogue number. If plant specimens were collected from the field, describe the collection location, date and sampling procedures.

Novel plant genotypes

Describe the methods by which all novel plant genotypes were produced. This includes those generated by transgenic approaches, gene editing, chemical/radiation-based mutagenesis and hybridization. For transgenic lines, describe the transformation method, the number of independent lines analyzed and the generation upon which experiments were performed. For gene-edited lines, describe the editor used, the endogenous sequence targeted for editing, the targeting guide RNA sequence (if applicable) and how the editor was applied.

Authentication

Describe any authentication procedures for each seed stock used or novel genotype generated. Describe any experiments used to assess the effect of a mutation and, where applicable, how potential secondary effects (e.g. second site T-DNA insertions, mosiacism, off-target gene editing) were examined.

Flow Cytometry

Plots

Confirm that:

☒ The axis labels state the marker and fluorochrome used (e.g. CD4-FITC).

☒ The axis scales are clearly visible. Include numbers along axes only for bottom left plot of group (a 'group' is an analysis of identical markers).

☒ All plots are contour plots with outliers or pseudocolor plots.

☒ A numerical value for number of cells or percentage (with statistics) is provided.

Methodology

Sample preparation

For cell lines or CD34+ cells or Mononuclear cells, cells were washed with PBS and counted before staining. Then, at least 1x10^5 cells were incubated for 30 minutes in a 37 °C cell culture incubator for dyes (namely for ROS; Mitochondrial potential; glutathione, GSH). After washing, where specified, the cells were stained with specific surface markers using antibodies detailed in Method section. Cells were incubated with the appropriate dilution of fluorescent antibody conjugates and were stained at room temperature for 15 minutes, washed with staining buffer, and analyzed using Cytoflex (Beckman Coulter) and Kaluza 2.1 software.

For fresh blood samples, based on White Blood Cell (WBC) counts, 50x10^4 cells (in whole blood) were incubated with dyes (namely ROS; Mito; GSH) for 30 minutes in a 37 °C cell culture incubator. For redox studies, the whole blood was lysed with BD FACS™ Lysing solution (BD Biosciences, San Jose, CA, USA) for 10 minutes at room temperature, washed with FACS buffer and lysed again for 5 minutes before staining for surface markers, as described above.

For SCENITH analysis, fresh whole blood (based on WBC counts) and MNCs (1 × 10^6) or CD34+ cells (2 × 10^5), harvested at desired time points were treated for 15 minutes with control (Co), 2-deoxy-glucose (2-DG; 100 mM), oligomycin (O; 1 µM), a combination of 2DG and oligomycin (DGO) or harringtonine (H; 2 µg/mL). Following metabolic inhibition, puromycin (final concentration 10 µg/mL) was added to the cultures for 40 minutes n a 37 °C cell culture incubator. After puromycin treatment, the cells were washed in cold PBS and stained with eBioscience™ Fixable Viability Dye eFluor™ 780 (Thermo Fisher Scientific, Waltham, MA, USA) for 15 minutes at 4°C in PBS. Then, cells were stained with primary antibodies against surface markers as reported in Method section, for 25 minutes at 4°C in FACS buffer. Cells were fixed and permeabilized using the Foxp3 Transcription Factor Staining Buffer Set (Thermo Fisher Scientific, Waltham, MA, USA) according to the manufacturer's instructions. Intracellular staining of puromycin was performed for 1 hour in diluted permeabilization buffer at 4°C. Finally, data acquisition was performed using the CytoFLEX flow cytometer or spectral flow cytometry.

For extracellular vesicle analysis, multiplex bead-based assay was performed using the MACSPlex Exosome Kit, human (Miltenyi Biotec, Bergisch Gladbach, Germany), according to the manufacturer's instructions. Briefly, solated extracellular vesicles were diluted in MACSPlex buffer with MACSPlex exosome capture beads at room temperature, overnight. Then, MACSPlex Exosome Detection Reagent for CD9, CD63, and CD81 was added to each well in 96well plate, followed by incubation for 1 hour at room temperature and washed. For analysis, median fluorescence intensity (MFI, APC) was evaluated for each capture bead subset and corrected by subtracting the respective MFI of the blank control (buffer+capture beads+antibodies without EVs).
The PKH67 Green fluorescent cell linker (Sigma–Aldrich, Milan, Italy) was used to label the EV membrane. The EV from AML patients were labeled with dye (1:80) for 5 min in Diluent C. After adding 1% bovine serum albumin (BSA) to quench the EVs, the samples were washed with serum-free media, and the redundant dye was removed by ultrafiltration twice with Ultra-15 centrifugal filters and Amicon Ultra-2 (Merck Life Science S.r.l., Milan, Italy) for 40 minutes. Then, PKH67-labeled EVAML were added to the cell culture with AML CD34+ cells or leukemia cell lines for up to 24 hours.

In a set of experiments, PB CD34+ AML cells that had been previously purified were thawed and prepared for FACS sorting. Sorting was performed using a 100 µm nozzle with a pressure of 20 PSI.

Instrument	CytoFLEX B5-R3-V5; SONY ID7000; 5L Cytek Aurora spectral cytometer (Cytek Bioscience); BD FACSAria™ Fusion Special Order (SORP) cell sorter cytometer
Software	Kaluza Analysis 2.1.
Cell population abundance	CD34+ cells (mean purity > 90%) were purified from AML MNCs and from cord blood units by immunomagnetic separation (Miltenyi Biotec, Bergisch Gladbach, Germany) according to the manufacturer's recommendations.
Gating strategy	Gating strategy varied according to the experiments anmd reported in Supplementary Figure 1a, e and Supplementary Figure 7c. In general, single cells taken from FSC-A vs FSC-H and assigned live or dead based on controls (unstained and single stained controls) and the relative gating was based on single stained positive versus negative controls for each individual fluorochrome or dye. For whole blood staining, the gating strategy was standardized and built considering the staining of CD3 + lymphocytes within each AML sample. Representative dot plots were inserted in Fig. 1a for the gating strategy to profile CD34+ cells based on CD3+ cells (as reference cells) for two-by-two staining combinations including CellROX (ROS), MitoTracker CMXRos (MITO) and Thiol Tracker (GSH).<br>Based on CD3+ cell staining for ROS/MITO, ROS/GSH and GSH/MITO, we used the following gates on:<br>-live singlets CD34+: ROShi/MITOlo < ROShi/MITOhi < ROSlo/MITOhi < ROSlo/MITOlo<br>-live singlets CD34+: ROShi/GSHlo < ROShi/GSHhi < ROSlo/GSHhi < ROSlo/GSHlo<br>-live singlets CD34+: GSHhi/MITOlo < GSHhi/MITOhi < GSHlo/MITOhi < GSHlo/MITOlo<br>For human cell lines and CD34+ cells, we developed a gating strategy combining side by side the different staining (for ROS, MITO and GSH) suitable among the cell lines and human primary cells CD34+ with/without extracellular vesicles.<br>Gating strategy 1. Blast populations were identified by CD45low/-/SSC gating strategy to define and analyze mainly CD34+ stem cells, immature CD34+CD38low/- stem cells and CD34+CD38+ progenitor cells. For gating strategy 2, blast populations were identified by CD45low/-/SSC gating strategy to define and analyze immature and progenitor leukemic cells (CD34+ and/or, CD117+), myeloid cells (CD33+, HLA-DR+), and primitive LSC (CD123+).

☒ Tick this box to confirm that a figure exemplifying the gating strategy is provided in the Supplementary Information.
